# A Review of Macrophage MicroRNAs’ Role in Human Asthma

**DOI:** 10.3390/cells8050420

**Published:** 2019-05-08

**Authors:** Gavriela Feketea, Corina I Bocsan, Cristian Popescu, Mihaela Gaman, Luminita A Stanciu, Mihnea T Zdrenghea

**Affiliations:** 1 Department of Hematology, Iuliu Haţieganu University of Medicine and Pharmacy, 400124 Cluj-Napoca, Romania; Fechetea.Gabriela@umfcluj.ro (G.F.); popescu.cristian@umfcluj.ro (C.P.); 2 Department of Pharmacology, Toxicology and Clinical Pharmacology, Iuliu Haţieganu University of Medicine and Pharmacy, 400337 Cluj-Napoca, Romania; bocsan.corina@umfcluj.ro; 3 Department of Hematology, Carol Davila University of Medicine and Pharmacy, 050474 Bucharest, Romania; mihaela_dervesteanu@yahoo.com; 4 National Heart and Lung Institute, Imperial College London, London W2 1PG, UK; 5 Department of Hematology, Ion Chiricuta Oncology Institute, 400010 Cluj-Napoca, Romania

**Keywords:** asthma, molecular asthma, macrophages, microRNA

## Abstract

There is an imbalance in asthma between classically activated macrophages (M1 cells) and alternatively activated macrophages (M2 cells) in favor of the latter. MicroRNAs (miRNAs) play a critical role in regulating macrophage proliferation and differentiation and control the balance of M1 and M2 macrophage polarization, thereby controlling immune responses. Here we review the current published data concerning miRNAs with known correlation to a specific human macrophage phenotype and polarization, and their association with adult asthma. MiRNA-targeted therapy is still in the initial stages, but clinical trials are under recruitment or currently running for some miRNAs in other diseases. Regulating miRNA expression via their upregulation or downregulation could show potential as a novel therapy for improving treatment efficacy in asthma.

## 1. Introduction

Although it was recently reported that there is a spectrum of human macrophage activation states, two main polarized states have been described for macrophages—pro-inflammatory classically activated M1 and anti-inflammatory alternatively activated M2 macrophages [1,2,3]. Interferon-γ (alone or in combination with microbial stimuli and/or inflammatory cytokines) induces M1 macrophages, and cytokines such as IL-4 and IL-13 induce the alternatively activated M2 macrophages. Macrophage functions depend on polarization status: M1 macrophages are characterized by a pro-inflammatory phenotype and functions, whereas M2 macrophages display anti-inflammatory functions [1]. It has been reported that resident macrophages nest in various tissues including the lungs. They express a number of M2 markers, including Ym1, IL-10, IL-4, CD206, Fizz1, and Arg1, suggesting that tissue-resident macrophages are already polarized towards an anti-inflammatory M2 phenotype under normal physiological conditions [4]. 

Allergic asthma is a chronic inflammatory disease characterized by an imbalance between exaggerated type 2 and deficient type 1 immune responses, including a predominant M2 macrophage phenotype [5]. At the moment, based on inflammatory cell count (eosinophilic, neutrophilic, and paucigranulocytic) in tissue and blood, two main subtypes of type 2 inflammation have been defined in asthma: T helper type 2 cell high (T2-high) and T helper type 2 cell low (T2-low). T2-high phenotypes have been classified into three groups: early-onset allergic asthma, late-onset eosinophilic asthma, and aspirin-exacerbated respiratory disease. The non-T2-high (T2-low) endotype is characterized by neutrophilic or paucigranulocytic (i.e., normal sputum levels of both eosinophils and neutrophils) inflammation and a lack of response to corticosteroid therapy [6,7]. In a recent report from the U-BIOPRED about an adult asthmatic study, four asthma phenotypes were identified: phenotype 1, moderate-to-severe asthma; and three severe asthma phenotypes, namely, phenotype 2 (T2) with severe late-onset eosinophilic asthma; phenotype 3 (T3) with severe asthma, oral corticosteroid therapy-dependent; and phenotype 4 (T4) with severe asthma, female predominance, obesity. The severe asthma clusters (T2 to T4) had higher sputum eosinophilia than cluster T1, with no difference in sputum neutrophil counts, exhaled nitric oxide and serum IgE levels, and received oral corticosteroids frequently [8].

The microRNA (miRNA) family is a class of small (20–30 nucleotides in length) non-protein-coding RNAs. MiRNA sequences are transcribed by RNA polymerase II into primary miRNAs (pri-miRNAs) that generate precursor miRNAs (pre-miRNAs) which are exported into the cytoplasm where they inhibit target gene expression by mRNA degradation and/or mRNA inhibition of translation to target protein. MiRNAs are abundant in the circulation and some have been proposed as useful tools for diagnosis, prognosis, and monitoring of treatment outcomes in cancer and other diseases. Deregulation of certain miRNAs is thought to play a role in asthma pathogenesis. Because miRNA functions demonstrated in animal studies may not reflect their role in human asthma, this review focuses mainly on miRNAs with relevance for human adult asthma [9,10,11]. 

Several miRNAs have been implicated in the regulation of macrophage activation and polarization through targeting adaptor proteins and transcription factors [12,13,14,15,16,17,18]. miRNAs have been found to characterize M1- or M2-polarized macrophages and, even more, to promote M1- or M2-polarization [16,18]. When miRNA expression profiles were determined in human monocyte-derived macrophages (MDMs) incubated in conditions causing activation toward M1- and M2-phenotypes, miRNA-29b-3p, miRNA-145-5p, miRNA-146a-5p, miRNA-147b, miRNA-155, miRNA-193a levels were increased in M1 cells (obtained by 5-days stimulation with IFN-γ + TNF-α) [15]. In M2a phenotype (obtained by stimulation with IL-4), miRNA-500a, miRNA-502, miRNA-511 were upregulated and miRNA-147b, miRNA-181a/b were down-regulated. In M2c phenotype (obtained by stimulation with IL-10) miRNA-21, miRNA-22, miRNA-27a, miRNA-146b-5p, were up-regulated and miRNA-155, miRNA-200a, miRNA-339 were downregulated [15].

## 2. MiRNAs Relevant for Asthma

Current reports about miRNA levels in asthma are contradictory, with some reporting upregulation and others reporting downregulation for the same miRNAs. It appears, however, that miRNA dysregulation could be associated with asthma pathogenesis and responsiveness to treatment in asthma. MiRNAs are present in lung cells (airway biopsies, epithelial brushings), sera/plasma, and other extracellular fluids (sputum, bronchoalveolar lavage (BAL), exosomes [19], exhaled breath condensate). Their levels could be measured and correlated with clinical, biological, and functional parameters in asthma. 

### 2.1. MiRNAs Polarizing Macrophages Towards a Pro-Inflammatory M1 Phenotype

**Figure 1:**
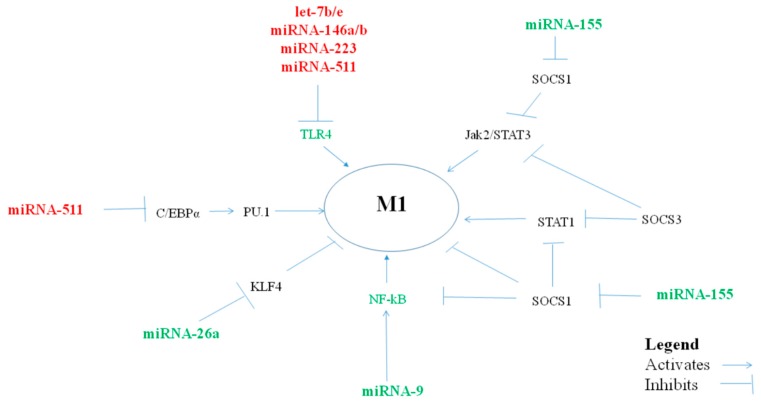
miRNAs, via their targets, influence pro-inflammatory M1-macrophage polarization.

The miRNAs that polarize macrophages towards a pro-inflammatory M1 phenotype (Table 1, Figure 1) are important in balancing the anti-inflammatory/regulatory M2 macrophages which promote Th2 immune responses. In addition, pro-inflammatory M1 macrophages promote T helper/cytotoxic type 1 antiviral responses, and virus infections are the most common cause of asthma exacerbations. An excessive macrophage pro-inflammatory response could, however, play a role in severe asthma and, particularly, in neutrophilic asthma. 

#### 2.1.1. MiRNA let-7f 

Let-7f miRNAs belong to a highly conserved let-7 (lethal-7) microRNA family consisting of 12 genes encoding for nine different miRNAs (let-7a to let-7i). Although the members share the same “seed region” (similarities in nucleotides 2–8 at their 5′ end), let-7 family members are each unique and encoded on different chromosomes [31]. Among the tumor suppressor miRNAs, reduced let-7 expression occurs most frequently in cancer and typically correlates with poor prognosis. 

Let-7f appears to polarize macrophages towards an M1 phenotype. 

In the THP1 cell line, pri-let-7f levels were increased in response to LPS while the corresponding mature miRNA let-7f levels were reduced. The overexpression of let-7f in THP-1 differentiated macrophages led to a reduction in SOCS4 protein levels [32].

When individual members of the let-7 family were measured, the expression of let-7f was reported to be higher in cultured epithelial brushings from asthmatic donors [20]. 

IL-17A is associated with severe asthma and requires IL-23R signaling, which is negatively regulated by let-7f miRNA in CD4+ lymphocytes [33]. Let-7f was negatively regulated by estrogen receptor signaling in the MCF-7 breast cancer cell line [34]. Women have an increased prevalence of severe asthma compared with men. In patients with severe asthma, IL-17A production was increased to a greater degree in TH17 cells from women compared with those from men [35]. Let-7f expression was lower and IL-23R expression was higher in TH17-differentiated cells from women compared with men with severe asthma [35].

#### 2.1.2. MiRNA-9 

Expression of miRNA-9 was found higher in pro-inflammatory M1 than M2 macrophages, and promoted human M1 polarization [2] and miRNA-9 levels correlated with levels of peroxisome proliferator-activated receptor-δ (PPAR-δ, also known as PPAR-β) in M1 macrophages [36].

MiRNA-9 levels were synergistically increased following IFNγ/LPS co-exposure in isolated macrophages and in vivo, in an IFN-γ/LPS–induced mouse model of steroid-resistant airway hyperresponsiveness (AHR) [21]. MiRNA-9 production decreased protein phosphatase 2A (PP2A) activity and also inhibited steroid-induced glucocorticoid receptor (GR) nuclear translocation. AntagomiRNA-mediated inhibition of miRNA-9 increased both PP2A activity and GR nuclear translocation in macrophages, and restored steroid sensitivity in isolated macrophages and in several mouse models of steroid-resistant AHR [21]. 

The pro-inflammatory miRNA-9 was found to be increased in sputum samples from patients with neutrophilic asthma, which is often resistant to corticosteroid therapy, compared with eosinophilic asthma [21]. Animal studies suggest that targeting miRNA-9 could be an effective treatment for steroid-resistant asthma.

#### 2.1.3. MiRNA-17-18-19-20-92 

The miRNA-17-92 cluster (miRNA-17-5p, miRNA-17-3p, miRNA-18a, miRNA-19a, miRNA-19b, miRNA-20a, and miRNA-92-1) or oncomiRNA-1 increases cell proliferation or inhibits apoptosis by suppressing pro-apoptotic Bcl2 protein Bim, transcription factor E2 factor 1 (E2F1), and tumor suppressor phosphate and tensin homolog (PTEN). Downregulation of miRNA-17-92 is critical for normal myeloid differentiation via induction of PU.1 [37]. PU.1 transcription factor has been reported to play an important role in the differentiation of macrophages towards the pro-inflammatory M1-like phenotype [38].

MiRNA-19 has a direct role in upregulating NF-*κ*B signaling and pro-inflammatory cytokine production. miRNA-19a-3p is capable of inducing an M1 phenotype by targeting Fos-related antigen-1 (Fra-1) transcription factor which plays a key role in the polarization of the M1 to the M2 phenotype and, consequently, decreasing the expression of the Fra-1 downstream genes VEGF, STAT3, and pSTAT3 [39]. 

The available studies concerning the levels of individual miRNAs of the miRNA-17-92 cluster in different biological specimens in asthma report decreased levels of miRNA-17, miRNA-18, miRNA 19b, and miRNA-20. On the contrary, one study reported increased miRNA-19a levels. **miRNA-17-5p** was reported downregulated in brushing bronchial epithelial cells cultured in vitro from severe asthmatics compared to cells from healthy donors [26]. Downregulation of **miRNA-18a** was observed in nasal mucosa biopsies in patients with asthma in comparison with control subjects [23], and in bronchial epithelial cells, obtained by brushing and cultured in vitro, from patients with asthma with different degrees of severity compared to cells from healthy donors [22]. Upregulation of **miRNA-19a** was found in epithelial cells isolated from biopsies of subjects with severe asthma (only 6) compared with cells from subjects with mild asthma (9) and healthy controls (9), and miRNA-19a was shown to enhance proliferation of bronchial epithelial cells from patients with severe asthma by targeting the TGF-β receptor 2 gene [24]. The levels of **miRNA-19b-3p** were found to be decreased in bronchial cells obtained by bronchoscopic brushing from patients with steroid-naïve asthma [25] and in brushing bronchial epithelial cells from severe asthmatics cultured in vitro compared to cells from healthy donors [26]. **miRNA-20a-5p** levels were found to be downregulated in brushing bronchial epithelial cells cultured in vitro from severe asthmatics compared to cells from healthy donors [26]. In another study, miRNA-20 was expressed at the same level in primary bronchial epithelial cells of patients with severe or difficult-to-treat asthma, treated with inhaled corticosteroids and subjects without asthma [40]. 

#### 2.1.4. MiRNA-26a/b

It was shown that miRNA-26a directly downregulated IFN-β in human macrophages [41]. miRNA-26a induced a pro-inflammatory M1-type of human macrophage activation by downregulating M2-polarizing macrophage colony-stimulating factor (M-CSF) and IL-10 expression [42]. Kruppel-like factor 4 (KLF4) was a target of miRNA-26a-5p in primary human macrophages [43].

All available studies report decreased levels of miRNA-26 in asthma. miRNA-26a has been found to be decreased in bronchial epithelial cells obtained by brushing from patients with steroid-naïve asthma [25]. Levanen and colleagues demonstrated for the first time the presence of miRNAs in exosomes from BAL fluid of both healthy and asthmatic subjects with mild intermittent stable disease [28]. They showed that exosomal miRNA profiles at baseline are different, and in asthma the expression profiles of the identified miRNAs were highly correlated with forced expiratory volume in 1 s (FEV1). Levels of miRNA-26a were downregulated in BAL fluid exosomes in patients with mild asthma [28]. Decreased expression of serum miRNA-26a was observed in a very small group of mild persistent or moderate persistent asthmatic patients (*n* = 10, 8/10 allergic, smokers 4/10), compared to control subjects (*n* = 10, 3 being allergic, smokers 5/10) [44]. In another study of the same group, the plasma levels of miRNA-26b, but not miRNA-26a, were decreased in patients with asthma [27].

#### 2.1.5. MiRNA-27a/b

In human MDM, miRNA-27a expression was higher in M2b macrophages [13]. Treatment with LPS, IFN-β, or IFN-γ repressed accumulation of miRNA-27a during human monocyte-to-macrophage differentiation (IFN-β more than IFN-γ) [45]. The stimulation through Toll-like receptor TLR2/TLR4 (not TLR3) decreased miRNA-27a in human MDM (monocytes were cultured with M-CSF). Upregulation of miRNA-27a enhanced the expression of pro-inflammatory cytokines in TLR2/4-activated macrophages. 

The expression levels of miRNA-27a and miRNA-27b-3p in freshly isolated bronchial epithelial brushings were lower in patients with steroid-naïve asthma and steroid-using asthma, compared to healthy control subjects [25]. miRNA-27a and miRNA-27b-3p expression was downregulated in bronchial epithelial cells obtained by brushing and cultured in vitro from patients with asthma with different degrees of severity, compared to cells obtained from healthy donors [22].

#### 2.1.6. MiRNA-125b

In vitro, miRNA-125b-5p was significantly upregulated in M1 and M2a/M2c-polarized macrophages compared with unpolarized macrophages [15]. MiRNA-125b was significantly upregulated by TLR4 engagement in THP-1 cells and miRNA-125b overexpression induced M1 polarization in THP-1 cells, mimicking the IFN-γ/LPS stimulation effect [46]. 

Circulating plasma miRNA-125b levels were most predictive of asthmatic status, being increased in patients with asthma who had high eosinophil counts [27].

#### 2.1.7. MiRNA-155

MiRNA-155 was increased in M1 and was downregulated in IL-10-polarized human M2c macrophages [2,15,47,48]. miRNA-155 promotes pro-inflammatory classical M1 activation by blocking anti-inflammatory signals and transcription factors, but it can also prevent excessive TLR signaling. Early during TLR activation, miRNA-155 expression is upregulated and inhibits the expression of the negative regulators, such as IL-10, allowing TLR signal transduction and type I IFN-mediated antiviral response. Later on, the increase in anti-inflammatory miRNA-21 induces IL-10 production, and increased IL-10 reduces miRNA-155 expression, limiting the TLR signaling pathways [49]. miRNA-155 also enhances M1-polarization by the repression of negative regulators of pro-inflammatory responses including SOCS1, SH2 domain-containing inositol 5-phosphatase 1 (SHIP1), and BCL6 in various diseases. The serine-threonine kinases Akt contribute to macrophage polarization, and miRNA-155 was found to be essential in serine-threonine kinase Akt–dependent M1/M2 polarization of macrophages (with Akt1 involved in M2- and Akt2 in M1-polarization) by targeting CCAAT/enhancer binding protein-β (C/EBPβ), a key regulator of M2 polarization [50]. SOCS2, a marker of M2, is an essential controller of macrophage activation and function, and also regulates SOCS1 and SOCS3 expression levels through proteasomal degradation [51]. 

In human macrophages, but not in BEAS-2B bronchial epithelial cell line, miRNA-155 downregulated the levels of IL-13Rα1, thus reducing the phosphorylation of STAT6 [12,52]. 

Data regarding the levels of miRNA-155 in asthma are divergent. In primary non-asthmatic and asthmatic human airway smooth muscle cells (hASMCs) isolated from non-transplantable donor lungs or resected lung tissue by enzymatic digestion, miRNA-155 expression was higher in IL-1β/TNF-α/IFN-γ-treated asthmatic cells as compared to normal cells [53]. 

Levels of miRNA-155 were lower in asthmatic bronchial epithelial cells obtained by brushing and cultured in vitro, than in cells from healthy donors [22]. miRNA-155 expression was also found downregulated in the nasal mucosa [23], in exhaled breath condensates [30], and in cell-free induced sputum in patients with asthma in comparison to control subjects [29]. Low levels of miRNA-155 were reported also in plasma of patients with asthma [27]. In a recent report, patients with severe asthma had higher plasma levels of miRNA-155 when compared with mild-to-moderate asthmatics and non-asthmatic control subjects, suggesting that miRNA-155 may contribute to the severity of inflammation [54].

Recent animal data showed increased expression of miRNA-155 in an ovalbumin (OVA)-induced mouse model of asthma, and lentiviral vector-delivered small interfering (si)RNA targeting miRNA-155 resulted in reduced AHR, airway inflammation, and Th2 cytokine production [55]. These data suggest that miRNA-155 could be involved in asthma severity and that targeting miRNA-155 could be a novel approach for the treatment of allergic asthma [55].

In conclusion, pro-inflammatory miRNA-9, miRNA-18/19, miRNA-26, miRNA-27, miRNA-125b, and miRNA-155 have been shown in vitro to polarize macrophages towards a pro-inflammatory M1 phenotype and their levels were generally found to be decreased in various tissues in asthma. Theoretically, M1-polarized macrophages balance M2 macrophages and play a positive role in both normal individuals and in patients with asthma. However, these pro-inflammatory miRNAs in excess may play a detrimental role in severe asthma. 

MiRNA-9 was increased in patients with neutrophilic asthma and the targeting of miRNA-9 has been suggested for the treatment of steroid-resistant asthma.

MiRNA-125b and miRNA-155 were increased in severe asthma, which would suggest that in this situation targeting these miRNAs could be an additional tool in its treatment.

### 2.2. MiRNAs Polarizing Macrophages towards an anti-Inflammatory/Suppressor M2 Phenotype

**Figure 2:**
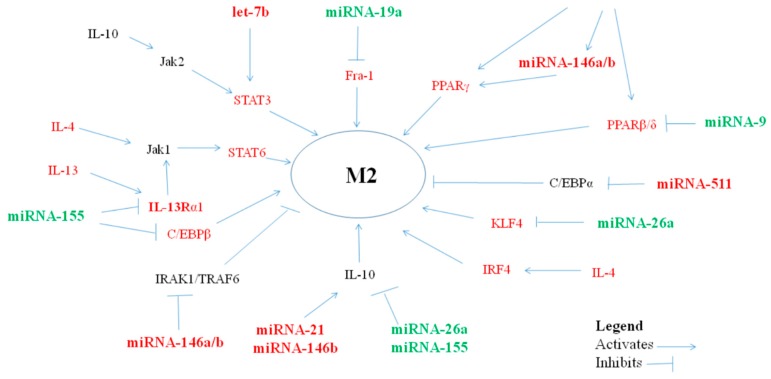
miRNAs, via their targets, influence anti-inflammatory M2-macrophage polarization.

Certain miRNAs may prove to play an important role in the pathogenesis of allergic inflammation in asthma by polarizing macrophages towards the M2 phenotype (Table 2, Figure 2). 

#### 2.2.1. MiRNA let-7a/b/c/d/e

The miRNA let-7 family members are generally implicated in regulating the TLR/NF-*κ*B signal pathway and are known as tumor suppressor miRNAs, participating in cell differentiation, proliferation, and apoptosis [61]. The levels of let-7a/b/c/e were found upregulated in tumor-associated macrophages (TAMs), which displayed characteristics of anti-inflammatory M2 macrophages [62]. 

Let-7 decreased the expression of TLR4 in various cell types [63,64].

Levels of **let-7a** were significantly decreased in exosomes isolated from BAL fluid in patients with mild intermittent asthma [28] and in exhaled breath condensates from patients with asthma [30]. Serum levels of miRNA-7a were reported to be decreased in patients with asthma [44].

In human macrophages, IFN-β upregulated the expression of **let-7b**, and let-7b directly downregulated IFN-β, suggesting a negative feedback loop [41]. LPS-preconditioned mesenchymal stromal cells containing exosome (extracellular vesicles derived from cell endocytosis which act as transmitters between cells)-shuttled let-7b polarized THP-1 cells to a M2 phenotype via TLR4/NF-*κ*B/STAT3/AKT regulatory signaling pathway [65]. The upregulation of let-7b is characteristic of prostatic TAMs. The downregulation of let-7b in TAMs leads to changes in the expression profiles of cytokines, such as those of IL-12, IL-23, IL-10, and TNF-α [62]. 

In contrast to reports of mouse studies, TLR7/TLR8 activation triggered monocytes to differentiate into a M2 macrophage surface phenotype with a mixed M1/M2 cytokine secretion profile [66].

Treatment with antagomiRNA against let-7b in a house dust mite-induced model of AHR did not suppress features of the disease [67].

Plasma let-7b expression was found increased in patients with asthma who had high eosinophil counts [27].

Levels of let-7c were found decreased in bronchial cells obtained by bronchoscopic brushing from patients with steroid-naïve asthma [25]. let-7c levels were reported increased in plasma in asthmatic patients with high eosinophil counts [27].

Serum miRNA let-7d levels were reported decreased in patients with asthma [44].

let-7e targeted STAT3 inhibitor SOCS1 in human suppressor antigen-presenting cells (obtained by monocyte stimulation with the TLR7/8- and NLR-activator R848 (Resiquimod)) [68]. In primary nasal epithelial cells from patients with allergic rhinitis, one pathway of the anti-inflammatory effect of miRNA-let-7e was via activation of the JAK1/STAT3 pathway, which caused a decrease in SOCS4 expression [69].

In human THP-1 cells, let-7e targeted TLR4 and pro-inflammatory cytokines, suggesting a feedback mechanism TLR4-let-7e-TLR4 [64]. 

In the nasal mucosa of patients with asthma, let-7e expression was lower than in control subjects [23], but plasma let-7e levels were reported increased in asthmatic patients with high eosinophil counts [27].

#### 2.2.2. MiRNA-21

In in vitro IL-10-stimulated macrophages (i.e., M2c macrophages), miRNA-21 was shown to be increased [15]. The miRNA-21-containing exosomes, engulfed in vitro by CD14^+^ human monocytes, suppressed the expression of M1 and increased that of M2 markers [70]. 

By targeting transcripts of proteins that regulate cell division and apoptosis, such as PTEN and programmed cell death 4 (PDCD4) (negative regulators of cell death and cell cycle, respectively) miRNA-21 is considered an oncomiRNA. miRNA-21 inhibited NF-*k*B and the production of IL-6 and increased IL-10 indirectly, via downregulation of the pro-inflammatory molecule tumor suppressor PDCD4, a negative regulator of AP-1 and IL-10, in a macrophage cell line and bone marrow-derived macrophages [71,72]. 

Upregulation of miRNA-21 was observed in diphenylcyclopropenone-challenged skin biopsies in human allergic contact dermatitis [73]. 

The data available concerning miRNA-21 levels in human adult asthma are conflicting, with different degrees of modulation being observed in different cell types and tissues. 

Levels of miRNA-21 were found decreased in exosomes from BAL and exhaled breath condensates from patients with asthma [28,30]. 

In contrast, miRNA-21 expression was increased in cultured epithelial cell specimens collected with a tracheal swab in patients with asthma regardless of treatment with inhaled corticosteroids (ICS) compared to the normal subjects, and was significantly higher in the ICS non-treated than the ICS-treated patients with asthma [56]. When these epithelial cells were cultured in vitro with IL-13, miRNA-21 expression increased with increasing IL-13 concentration. 

Upregulation of plasma miRNA-21 was also reported in eosinophilic asthma, and it was suggested that miRNA-21 represents a profile of type 2 airway inflammation [27]. 

The miRNA-21 seems to reflect the type 2 environment characteristic to asthma, and targeting miRNA-21 could be beneficial in asthma [56]. 

#### 2.2.3. MiRNA-34 

The miRNA-34 family is comprised of miRNA-34a, -34b, and -34c, of which miRNA-34a was the first identified tumor suppressor gene [74] and was shown to promote M2 polarization in vitro [2]. The expression of miRNA-34a-5p was found upregulated during in vitro maturation of monocytes to macrophages (unpolarized macrophages, M1, M2a, and a little more in M2c) compared with monocytes [15].

Anti-inflammatory miRNA-34a directly modulated IFN-β genes, pathways, and protein expression in human cells [41,75]. In primary primate macrophages, miRNA-34 reduced IFN-β protein production, and there is an apparent negative feedback loop, with increased expression of miRNA-34 in primate macrophages exposed to recombinant IFN-β or stimulated to produce IFN-β [41]. In addition, miRNA-34a promotes tumor suppressor protein TP53 (p53) expression by targeting the anti-aging factor Sirtuin-1 (SIRT1), a negative regulator of p53 [76], and p53 drives transcription of the miRNA-34 family, which activates apoptotic pathways.

Overexpression of miRNA-34a in human HL-60 and Kasumi-1 cells blocks PD-L1 expression, and reduces PD-L1 surface expression [77] via p53 [78]. Surface expression of PD-L1 induced by IFN-γ was also reversed by miRNA-34a. miRNA-34a transfection reduced the production of PD-L1 downstream of IL-10 and decreased PD-L1-specific T cell apoptosis [77]. 

No data are available on miRNA-34a in asthma, but miRNA-34b-5p and miRNA-34c-5p levels in freshly isolated bronchial epithelial brushings were lower in patients with steroid-naïve and steroid-using asthma, compared with healthy control subjects [25]. IL-13 stimulation in vitro decreased levels of miRNA-34b-5p and miRNA-34c-5p in human bronchial epithelial cells (obtained from airways of three lungs donated for lung transplantation and cultured at air–liquid interface (ALI) for 21 days) [25]. Serum levels of miRNA-34 were decreased in patients with asthma, concurrently with the upregulation of autophagy-related proteins and increased levels of pro-inflammatory factors and fibrosis-related proteins [57]. 

#### 2.2.4. MiRNA-124

MiRNA-124 was found increased in M2b-polarized macrophages, and miRNA-124 induced M2 polarization in macrophages by targeting various transcription factors and adaptor proteins [2]. 

Overexpression of miRNA-124 induced M2 polarization in porcine alveolar macrophages [79].

There are three subsets of monocytes in circulation: classical CD14^++^CD16^−^, with intermediate levels of miRNA-124; non-classical CD14^low^CD16^+^ which are miRNA-124-negative; and CD14^+^CD16^+^ intermediate monocytes containing the highest levels of miRNA-124. CD14^+^CD16^+^ (also referred to as CD14^++^CD16^+^ ) intermediate monocytes, which expressed high levels of miRNA-124 and exhibited other properties of M2-like cells (M2 surface markers such as CD163, CX3CR1, IL-4R, TGF-β1, and IL-10) [47] have been reported to be increased in patients with severe asthma compared to mild/moderate asthmatics [80]. MiRNA-124 is a potential therapeutic target in attempts to alter M1/M2 equilibrium in asthma.

#### 2.2.5. MiRNA-146a/b

MiRNA-146a and miRNA-146b expression was induced by TLR ligands and pro-inflammatory cytokines, and miRNA-146a induction was NF-*k*B-dependent [14,81]. 

Although miRNA-146a was increased in human M1-, and miRNA-146b in M2c-polarized macrophages [15], by negative feedback, both miRNA-146a and miRNA-146b are anti-inflammatory miRNAs, inhibiting NF-*k*B activation, and decreasing innate IFNs and pro-inflammatory cytokine production [82]. They act as a molecular brake, by targeting TNF receptor-associated factor 6 (TRAF6) and IL-1 receptor-associated kinase 1 (IRAK-1), key adaptor proteins of the NF-*k*B signaling pathway [82]. 

**MiRNA-146a** was reported to be increased in in vitro human M1-polarized macrophages [15]. A negative correlation was observed between miRNA-146a and genes involved in the inflammatory response, including TLR4, IRAK-1, IRAK-2, TRAF6, and IRF3 [83,84]. 

Higher miRNA-146a expression inhibited the proliferation and promoted apoptosis of human bronchial smooth muscle cells (BSMCs) in vitro by decreasing epidermal growth factor receptor (EGFR) expression, by reducing phosphorylated (p)-EGFR, p-extracellular signal-regulated kinase, p-stat3, and Bcl-2 expression, and by increasing caspase 3/7 activity [85]. miRNA-146a-5p was shown to suppress NF-*κ*B-inducible cytokine, CCL20, in airway smooth muscle cells (ASMCs) of patients with asthma and healthy individuals [58]. 

In primary non-asthmatic and asthmatic hASMCs isolated from non-transplantable donor lungs or resected lung tissue by enzymatic digestion, no differences in basal miRNA-146a or miRNA-146b expression were observed between asthmatic and non-asthmatic cells at 0 or 20 hours [86]. Differences in expression between non-asthmatic and asthmatic cells were evident when the cells were treated with the IL-1β/TNF-α/IFN-γ multicytokine cocktail/cytomix that mimics the inflammatory milieu [86]. Induction with cytomix was greater than induction with individual cytokines, and asthmatic ASMCs exhibited higher levels of miRNA-146a expression following IL-1β/TNF-α/IFN-γ cytomix treatment than non-asthmatic cells [86]. In another study, however, IL-1β induced lower miRNA-146a expression in ASMCs derived from patients with asthma compared with control subjects [58]. 

Williams and colleagues found no differences in miRNA-146a or miRNA-146b expression in airway biopsies from patients with mild asthma compared with control subjects [87]. However, in a recent study, decreased miRNA-146a expression was reported in vivo in bronchial biopsies from patients with asthma compared with healthy donors [58].

MiRNA-146a levels were reported to be increased in adults with asthma [27], and associated with indices related to worse asthma outcomes, including elevated blood eosinophil counts, higher asthma control questionnaire scores, and need for higher doses of ICS [88]. Overexpression of miRNA-146a in A549 human lung carcinoma cells combined with dexamethasone had greater anti-inflammatory effects than either alone, suggesting that miRNA-146a could be used to boost the anti-inflammatory effects of glucocorticosteroids in steroid-insensitive asthmatics [88].

**MiRNA-146b** is an anti-inflammatory tumor suppressor miRNA induced by TNF-α, IL-1β, and TLR agonists TLR2/4/5/7/8/9 but not TLR3 [81,89]. MiRNA-146b in human primary monocytes is expressed with delayed kinetics as compared to miRNA-146a, and was proposed to be a molecular effector of the IL-10 anti-inflammatory activity in monocytes [14]. 

Negative correlation was demonstrated between miRNA-146 expression and that of genes involved in immunity and inflammation (e.g., IRF7 and TLR4). MiRNA-146b modulates the TLR4 signaling pathway by direct targeting of multiple elements, including the LPS receptor TLR4 and the key adaptor/signaling proteins such as myeloid differentiation primary-response protein 88 (MyD88), IRAK-1, and TRAF6 [14,83]. The enforced expression of miRNA-146b in human monocytes led to a significant reduction in the TLR (LPS)-dependent production of several pro-inflammatory cytokines and chemokines [14]. In normal non-transformed cells, NF-*k*B activation increased STAT3 and consequently miRNA-146b which, as an endogenous negative feedback regulator, attenuated the activation of NF-*k*B and decreased STAT3 activation [90,91]. 

There are no studies reporting levels of miRNA-146b-5p in adults with asthma. 

#### 2.2.6. MiRNA-223-3p

As monocytes differentiate into macrophages, miRNA-223 expression decreases [15,92]. In vitro differentiation of human monocytes into macrophages with GM-CSF (or M-CSF) was accompanied by a decrease in the expression of miRNA-223, which led to a significant increase in the serine-threonine kinase IKKα protein expression (not of IKKβ or IKKγ). 

MiRNA-223 can regulate macrophage function by downregulating the canonical NF-*κ*B pathway [92]. MiRNA-223 promoted human M2 macrophage polarization [2] via targeting SOCS1, C/EBPβ, and PPAR-γ. 

In addition of macrophage regulation, miRNA-223 controls also eosinophils and neutrophils [93]. The upregulation of miRNA-223 was observed in diphenylcyclopropenone-challenged skin biopsies in human allergic contact dermatitis [73]. The most abundant miRNA in freshly isolated human neutrophils was miRNA-223 [94]. Neutrophil transfer of miRNA-223 to lung epithelial cells was reported [95], and miRNA-223 was expressed in cultured human bronchial epithelial cells, where it also downregulates NF-*κ*B signaling [96]. The administration of miRNA-223 mimics could thus restrict the migration of inflammatory cells to the site of infection and thereby diminish inflammation.

The expression of miRNA-223-3p was significantly upregulated in the sputum of patients with severe asthma compared with that in healthy control subjects, and miRNA-223-3p expression was associated with sputum neutrophilia and also with airway obstruction (FEV1/forced vital capacity) [59]. Plasma miRNA-223 levels have been reported to be increased in patients with asthma [27]. 

#### 2.2.7. MiRNA-511-3p

MiRNA-511-3p is transcriptionally coregulated with mannose receptor C-type 1 (Mrc1), which tends to polarize macrophages towards M2 phenotypes, and regulates the activation of macrophages [60,97]. miRNA-511 displayed low levels in monocytes, M1, and M2c and was highly expressed in M2a macrophages [15].

MiRNA-511-3p overexpression suppressed M1 with reduced expression of IL-1β, IL-6, and iNOS, and promoted M2 macrophage polarization with enhanced expression of Fizz1, Ym1, and Arg1. Potential targets of miRNA-511-3p include prostaglandin D2 synthase (PTGDS), ROCK2, LTBP1, and CCL2 as direct targets and TLR4 and C/EBPα as indirect targets [98]. 

It was reported recently that pro-inflammatory stimuli LPS and IFN-γ repressed miRNA-511-5p expression in human monocytes. IL-4 (and other stimuli inducing M2-like polarization, including IL-13, TGF-β, and Dexamethasone) induced the expression of mature miRNA-511-5p with similar kinetics to its host gene *MRC1*, coding for the prototypic M2 macrophage marker *Mrc1* (also known as CD206) [11]. TGF-β and glucocorticoids increased miRNA-511-5p expression, which then mediated glucocorticoid- and TGF-β-dependent hyporesponsiveness to endotoxin by direct targeting of TLR4 [11]. miRNA-511-5p acted as an intracellular mediator of glucocorticoid- and TGF-β-induced endotoxin tolerance in human monocytes through the direct and selective targeting of TLR4. 

Plasma miRNA-511-3p levels were significantly lower in patients with allergic asthma compared with those with allergic rhinitis, and with subjects without asthma or allergy [60].

In conclusion, the anti-inflammatory miRNAs have been shown in vitro to polarize macrophages towards an anti-inflammatory M2 phenotype. M2 macrophages, together with other type 2 cells, have an important role in allergic asthma. However, the published data concerning the levels of anti-inflammatory miRNAs in asthma are divergent. This could be attributed to few studies, heterogeneous asthma groups, different asthma phenotype, medication received, etc. It is only in recent years that asthma phenotypes have been reported, and criteria to identify them have been clarified. We also do not know yet how different medication alters miRNA levels in different biological samples. 

In one study, the levels of let-7b, let-7c, let-7e, miRNA-21, miRNA-146a, and miRNA-223 were found to be increased in plasma in patients with eosinophilic asthma. In another study of patients with severe asthma, miRNA-223 was reported increased in sputum (where macrophages are the predominant type of cells) and correlated with neutrophil counts. 

## 3. Discussions: microRNAs as Diagnostic Markers and Therapeutic Targets

There is an imbalance in allergic asthma between pro-inflammatory type 1 cells (T cells, B cells, dendritic cells, macrophages, etc.) and anti-inflammatory type 2 cells, in the favor of type 2 cells. Through their involvement in the differentiation, activation, and polarization of monocytes/macrophages, miRNAs could play an important role in the pathogenesis of asthma. Alteration of miRNA levels in macrophages should thus be able to influence the switch towards M1 and M2 phenotypes and between subtypes. 

As presented above, according to the current available studies, some miRNAs (i.e., miRNA-9, miRNA-27, miRNA-155, and miRNA-125b) can promote M1 polarization, while other anti-inflammatory miRNAs (miRNA-21, miRNA-223, miRNA-34, let-7c, miRNA-146a, and miRNA-511) can promote anti-inflammatory responses and M2 polarization in macrophages, in both circulatory monocytes and tissue-resident macrophages. The data available on miRNA levels in human adult asthma are conflicting, with different modulation demonstrated in different cell types and tissues. Their levels have been found to be either increased or decreased in various tissues in asthma.

The hypothesis that M1-promoting miRNAs would be decreased and M2-promoting miRNAs would be increased in asthma is not supported by the current data. It is known, however, that the M1 and M2 phenotypes are not inflexible, but rather reversible phenotypes. Plasticity is a fundamental characteristic of macrophages, and M1 and M2 are two extremes in a continuum of the polarization state [17].

Further study of miRNAs in polarized macrophages could help to identify specific miRNAs as markers for M1 or M2 macrophages, and miRNAs could be also proved to be valuable biomarkers for asthma diagnosis and assessment of asthma severity, and to help to discriminate between different asthma phenotypes, eosinophilic or neutrophilic. They may also be a useful adjuvant in asthma therapy and the monitoring of treatment. 

Specifically, certain miRNAs which have been found to correlate with disease severity could be proved to be useful as diagnostic biomarkers for asthma [99]. 

miRNAs could also be used to identify eosinophilic and neutrophilic asthma. Circulating miRNAs levels and, in particular, the plasma level of miRNA-125b, miRNA-21, and let-7b/c/e appear to be predictive of asthmatic status, being increased in patients with asthma who have high eosinophil counts [27]. The pro-inflammatory miRNA-9 was increased in sputum samples from patients with neutrophilic asthma, which is often more resistant to corticosteroid therapy than eosinophilic asthma, and animal studies suggest that targeting miRNA-9 could be effective for the treatment of steroid-resistant asthma [21].

Because of the M2/M1 imbalance in asthma, repolarizing M2 to M1 macrophages with miRNAs could be a possible approach for asthma treatment. Nevertheless, how to achieve macrophage-targeted miRNA delivery constitutes a challenge.

The expression of miRNAs can be controlled by miRNA inhibitors (antagomiRNA) or miRNA agonists (miRNA mimics). The knockdown of miRNAs is an effective method to use for characterizing the functions of miRNAs in vivo. AntagomiRNAs are chemically modified oligonucleotides complementary to individual miRNAs used to transiently inhibit miRNA function. The antisense oligonucleotide binds to the corresponding miRNA and blocks the interaction with its target rather than inducing its degradation.

miRNA-21 appears to reflect the type 2 environment characteristic of asthma, and targeting miRNA-21 could be beneficial in asthma therapy [56]. In a mouse model of acute bronchial asthma, miRNA-21 antagomiRNAs suppressed the development of allergic airway inflammation, inhibiting Th2 activation [100]. 

The pro-inflammatory miR-125b and miR-155 were observed to be increased in severe asthma, which would suggest that, in this situation, targeting these miRNAs could be an added tool in the treatment of severe asthma [55]. 

Earlier studies have identified miRNA-155 as a crucial positive regulator of Th1 immune response in autoimmune diseases, but also as a suppressor of Th2 immunity in allergic disorders. 

More recent studies provided new evidence that miRNA-155 plays an indispensable role in allergic asthma. When miRNA-155-5p was targeted by using a specific antagomiRNA in an OVA-induced allergic airways disease model [101], the antagomiRNA failed to alter the disease phenotype, although it effectively targeted myeloid cell populations. The author explained the failure of this miRNA-155-5p antagomiRNA to be due to limited ability to target lung lymphocytes. In another OVA-induced mouse model of acute asthma animal study, where miRNA-155 was upregulated, the inhibition of miRNA-155 using a lentiviral vector-delivered small interfering (si)RNA targeting miRNA-155 alleviated airway inflammation, AHR, and Th2 cytokine release [55]. The success of the lentiviral vector-mediated gene delivery system is explained by the high efficiency of gene transduction into a wide variety of dividing and non-dividing cells, and by long-term infection [55]. 

In an ex vivo animal study, the researchers generated redox/pH dual-responsive hybrid polypeptide nanovectors, which consisted of self-crosslinked redox-responsive nanoparticles based on galactose-functionalized n-butylamine-poly(l-lysine)-b-poly(l-cysteine) polypeptides (GLC) coated with DCA-grafted sheddable PEG-PLL (sPEG) copolymers [102]. Encapsulation with sPEG/GLC nanovectors effectively facilitated macrophage-targeted miRNA delivery in the acidic state, but diminished miRNA uptake at neutral pH. Administration of miRNA-155-loaded sPEG/GLC (sPEG/GLC/155) nanocomplexes increased miRNA-155 expression in TAMs about 100- to 400-fold, both in vitro and in vivo, and repolarized immunosuppressive TAMs to anti-tumor M1 macrophages through elevating M1 macrophage markers (IL-12, iNOS, and MHC II) and suppressing M2 macrophage markers (Msr2 and Arg1) in TAMs. Macrophage-targeted delivery of miRNAs with redox/pH dual-responsive sPEG/GLC nanovectors thus holds promise as an approach to repolarize M2 to M1 macrophages in situ [102].

A first-in-human phase 1 clinical trial of cobomarsen, an oligonucleotide inhibitor of miRNA-155 (antimir-155, MRG-106, miRagen Therapeutics) in patients with certain lymphomas and leukemias is currently underway to measure the safety, tolerability, and pharmacokinetics of this miRNA-155 inhibitor [103].

In another study, it was suggested that miRNA-146a could be used to boost the anti-inflammatory effects of glucocorticosteroids in steroid-insensitive asthmatics [88].

Recently, it has been shown that anti-inflammatory miRNA-125b can reprogram M2 into a pro-inflammatory (M1) phenotype [104]. Following intraperitoneal administration, the CD44 targeting hyaluronic acid-poly(ethylenimine) (HA-PEI)-based nanoparticles encapsulating miRNA-125b nanoparticles were shown to accumulate in the macrophage-ablated lung tissues of both naïve and KRAS/p53 double mutant genetically engineered (KP-GEM) non-small cell lung cancer (NSCLC) mouse model. In addition, a more than 6-fold increase in the M1 to M2 macrophage ratio was observed as well as a 300-fold increase in the iNOS (M1 marker)/Arg-1 (M2 marker) ratio in TAMs, compared to the untreated control group. Successful pro-tumor (M2) phenotype repolarization towards the M1 phenotype has significant implications in anticancer immunotherapy and, by association, for asthma.

## 4. Conclusions

Macrophage polarization is a highly plastic physiological process that responds to a variety of factors, by changing macrophage phenotype and function. Human data concerning miRNAs as modeling elements of the macrophage phenotype are frequently contradicted, however, by the in vivo data obtained from animal studies. Although the initial studies show promising results, further studies are required to elucidate the role of lung macrophages as a cellular source of miRNAs in the lung in asthma and to determine whether they may serve as biomarkers and potential therapeutic targets.

## Figures and Tables

**Table 1:** Pro-inflammatory miRNA levels in asthma.

miRNA	Macrophage Type	miRNA Levels in Asthma						
		Polarization	Serum/Plasma	Brushing Airway Cells	Biopsies	Nasal Mucosa	Sputum	BAL Exosomes/Exhaled Breath
let-7f	M1/M2a	M1/M2		↑^[20]^				
miRNA-9	M1	M1					↑^[21]^	
miRNA-18a	M1	M1		↓^[22]^		↓^[23]^		
miRNA-19a	M1	M1			↑^[24]^			
miRNA-19b-3p	M1	M1		↓^[25,26]^				
miRNA-26a/b	M2c	M1	↓^[27]^	↓^[25]^				↓^[28]^
miRNA-27a/b	M2b	M1		↓^[22,25]^				
miRNA-125b	M1/M2a/M2c	M1	↑^[27]^					
miRNA-155	M1	M1	↓^[27]^			↓^[23]^	↓^[29]^	↓^[30]^

**Table 2:** Anti-inflammatory miRNA levels in asthma.

miRNA	Macrophage Type	miRNA Levels in Asthma							
		Polarization	Serum/Plasma	Brushing Airway Cells	Biopsies	Nasal Mucosa	Sputum	BAL Exosomes/Exhaled Breath	Monocytes
let-7a	M2	M2	↓^[44]^					↓^[28,30]^	
let-7b	M2	M2	↑^[27]^						
let-7c	M2a	M2	↑^[27]^	↓^[25]^					
let-7e	M2	M2	↑^[27]^			↓^[23]^			
miRNA-21	M2c	M2	↑^[27]^	↑^[56]^				↓^[28,30]^	
miRNA-34	M2c	M2	↓^[57]^	↓^[25]^					
miRNA-124a	M2b	M2							↑^[47]^
miRNA-146a	M1	M2	↑^[27]^		↓^[58]^				
miRNA-223		M2	↑^[27]^				↑^[59]^		
miRNA-511	M2a	M2	↓^[60]^						
